# Risk factors for osteoradionecrosis of the jaw in patients with head and neck squamous cell carcinoma

**DOI:** 10.1186/s13014-020-01701-5

**Published:** 2021-01-05

**Authors:** Hikaru Kubota, Daisuke Miyawaki, Naritoshi Mukumoto, Takeaki Ishihara, Megumi Matsumura, Takumi Hasegawa, Masaya Akashi, Naomi Kiyota, Hirotaka Shinomiya, Masanori Teshima, Ken-ichi Nibu, Ryohei Sasaki

**Affiliations:** 1 Division of Radiation Oncology, Kobe University Hospital, Kobe, Hyogo Japan; 2 Department of Oral and Maxillofacial Surgery, Kobe University Graduate School of Medicine, Kobe, Japan; 3 Kobe University Hospital Cancer Center, Kobe, Japan; 4 Department of Otolaryngology-Head and Neck Surgery, Kobe University Graduate School of Medicine, Kobe, Japan

**Keywords:** Osteoradionecrosis of the jaw, Head and neck squamous cell carcinoma, Dose-volume histogram

## Abstract

**Background:**

To evaluate factors associated with osteoradionecrosis of the jaw (ORNJ) in patients with head and neck squamous cell carcinoma (HNSCC), focusing on jaw-related dose–volume histogram (DVH) parameters.

**Methods:**

We retrospectively reviewed the medical records of 616 patients with HNSCC treated with curative-intent or postoperative radiation therapy (RT) during 2008–2018. Patient-related (age, sex, history of smoking or alcohol use, diabetes mellitus, performance status, pre-RT dental evaluation, pre- or post-RT tooth extraction), tumor-related (primary tumor site, T-stage, nodal status), and treatment-related (pre-RT surgery, pre-RT mandible surgery, induction or concurrent chemotherapy, RT technique) variables and DVH parameters (relative volumes of the jaw exposed to doses of 10 Gy–70 Gy [V10–70]) were investigated and compared between patients with and without ORNJ. The Mann–Whitney U test was used to compare RT dose parameters. Univariate and multivariate Cox regression analyses were used to assess factors associated with ORNJ development. Kaplan–Meier analyses were performed for cumulative ORNJ incidence estimation.

**Results:**

Forty-six patients (7.5%) developed ORNJ. The median follow-up duration was 40 (range 3–145) months. The median time to ORNJ development was 27 (range 2–127) months. DVH analysis revealed that V30–V70 values were significantly higher in patients with than in those without ORNJ. In univariate analyses, primary tumor site, pre-RT mandible surgery, post-RT tooth extraction, and V60 > 14% were identified as important factors. In multivariate analyses, V60 > 14% (*p* = 0.0065) and primary tumor site (*p* = 0.0059) remained significant. The 3-year cumulative ORNJ incidence rates were 2.5% and 8.6% in patients with V60 ≤ 14% and > 14%, respectively (*p* < 0.0001), and 9.3% and 1.4% in patients with oropharyngeal or oral cancer and other cancers, respectively (*p* < 0.0001).

**Conclusions:**

V60 > 14% and oropharyngeal or oral cancer were found to be independent risk factors for ORNJ. These findings might be useful to minimize ORNJ incidence in HNSCC treated with curative RT.

## Background

Osteoradionecrosis of the jaw (ORNJ) is among the most serious late complications observed in patients with head and neck squamous cell carcinoma (HNSCC) treated with radiation therapy (RT). While ORNJ was first described in 1922, the definition, mechanism of pathogenesis, incidence, risk factors, and clinical staging, as well as treatment protocols associated with the disease require further investigation [1]. Despite the lack of a standard and unified definition, ORNJ is usually defined as an area of exposed irradiated bone that fails to heal over a period of 3–6 months in the absence of local tumor recurrence [2]. Radiological evidence of bone necrosis within the target volume is also important for the diagnosis and classification of ORNJ severity [3]. Several risk factors of ORNJ have been reported, including patient-related [4–7], tumor-related [4, 5, 8, 9], and treatment-related factors [5, 8, 10–16].

The reported incidence of ORNJ ranges widely according to the examined periods. The incidence of ORNJ has decreased in recent times, from approximately 20% several decades ago to 4–8% in the modern era [8, 13, 14, 17–19]. This tendency might be attributed to improvements in RT techniques, such as the intensity-modulated RT (IMRT), currently used. Several reports have indicated the trend in the rate of jaw-related complications among patients receiving IMRT compared with those receiving 3-dimensional conformal RT (3D-CRT) [11, 15, 20]. In contrast, no reductions were observed in long-term ORNJ rates following IMRT in the absence of attempts to reduce the jaw volumes receiving high doses in IMRT plan optimization [21]. Thus, it is necessary to determine the dose–volumetric threshold of the jaw for IMRT plan optimization to reduce the incidence of ORNJ. Nevertheless, few studies have reported the RT dose–volume correlation of the irradiated jaw [14–16], and the dose–volumetric threshold of ORNJ has not been clearly determined.

The purpose of this study was to identify the risk factors of ORNJ in patients with HNSCC in the modern era, including IMRT. Of the treatment-related factors, the dose–volume histogram (DVH) parameters of the jaw were intensively evaluated with the goal of achieving optimal dose constraints of the jaw and guiding the formulation of future planning objectives.

## Methods

### Patient selection

A total of 616 patients who received definitive-intent or postoperative RT to the head and neck between January 2008 and August 2018 at our institution were included in this retrospective analysis. Data were obtained from patients’ medical records, and the inclusion criteria were as follows: age over 20 years, histological confirmation of squamous cell carcinoma, follow-up duration of at least 3 months after RT, completion of the planned RT dose, no previous RT of the head and neck region, and presence of sufficient treatment RT plan data for the evaluation of the dose to the jaw. In this study, HNSCC included nasopharyngeal cancer, oropharyngeal cancer, hypopharyngeal cancer, oral cancer, and cervical lymph node metastases from unknown primary tumors. Patients with laryngeal cancer were not included in this study because the radiation fields for T1-2N0 laryngeal cancer are located completely outside the jaw.

### Treatment for HNSCC

Patients were treated with 3D-CRT (from 2008 to 2014) or IMRT (from 2014 to 2018). Computed tomography (CT) simulation was performed in patients immobilized using a thermoplastic mask. IMRT treatment typically included volumetric-modulated arc therapy using the simultaneous integrated boost (SIB-VMAT) technique. In general, 69.96 or 70 Gy (delivered in 2.12 Gy or 2 Gy per fraction) was prescribed for definitive RT, 50–60 Gy (delivered in 2 Gy per fraction) was prescribed for postoperative RT in cases with minor risk features, and 66 Gy (delivered in 2 Gy per fraction) was prescribed for postoperative RT in patients with high risk features. Dose to the jaw was not considered during RT planning. In general, tri-weekly cisplatin (dose 80–100 mg/m^2^) was used for concurrent chemoradiotherapy as part of both the definitive and postoperative therapies, while a few patients received concurrent cetuximab, as appropriate. Induction regimens administered to a small number of patients, as needed, also included docetaxel and/or fluorouracil. Before treatment, patients generally underwent pre-RT dental evaluation and management, including tooth extractions, as deemed appropriate by the oral and maxillofacial surgeons, based on risk assessment.

### Patient follow-up

After completing the RT, all patients were evaluated using a video laryngoscope every month for the 1st year, every 2 months for the next 2 years, and every 3 months thereafter for a total of at least 5 years by both radiation oncologists and head and neck surgeons. For oral care and dental evaluation, the patients were generally followed up at 3 and 6 months and at 1, 1.5, and 2 years after completing the RT at the Department of Oral and Maxillofacial Surgery of our hospital. If dental and oral symptoms, such as pain, trismus, and infection, were recorded during the follow-up period, they were initially treated conservatively. However, when the symptoms did not resolve and extraction was the only treatment option, dental extraction was considered.

### Definition of ORNJ

ORNJ was defined as an area of clinically exposed necrotic bone that was present in the radiation fields over a period of 3 months and/or required treatment with surgical intervention or hyperbaric oxygen therapy (HBO) without evidence of tumor recurrence [2]. There is also a subset of ORNJ that presents with clinically intact mucosa along with radiographic evidence including evidence derived from CT [3, 13, 22, 23]. We included both subsets in our cohort. ORNJ was graded according to the Common Terminology Criteria for Adverse Events v5.0 (CTCAE) [24]. The grades of ORNJ, as defined and graded according to the CTCAE v5.0, include grade 1: asymptomatic; clinical or diagnostic observations only; intervention not indicated; grade 2: symptomatic; medical intervention indicated (e.g., topical agents); limitations in the performance of instrumental activities of daily living (ADLs); grade 3: severe symptoms; limitations in the performance of self-care ADLs; elective operative intervention indicated; grade 4: life-threatening consequences; urgent intervention indicated; and grade 5: death. This study evaluated patients with grade ≥ 2 ORNJ.

### Potential risk factors of ORNJ

The potential risk factors of ORNJ included patient-related variables (age, sex, history of smoking, history of alcohol use, diabetes mellitus as a comorbidity, performance status, pre-RT dental evaluation, pre-RT tooth extraction, post-RT tooth extraction), tumor-related variables (primary tumor site, T-stage, nodal status), and treatment-related variables (pre-RT surgery, pre-RT mandible surgery, induction chemotherapy, concurrent chemotherapy, RT technique), and DVH parameters of the jaw.

### DVH analysis of the jaw

**Fig. 1:**
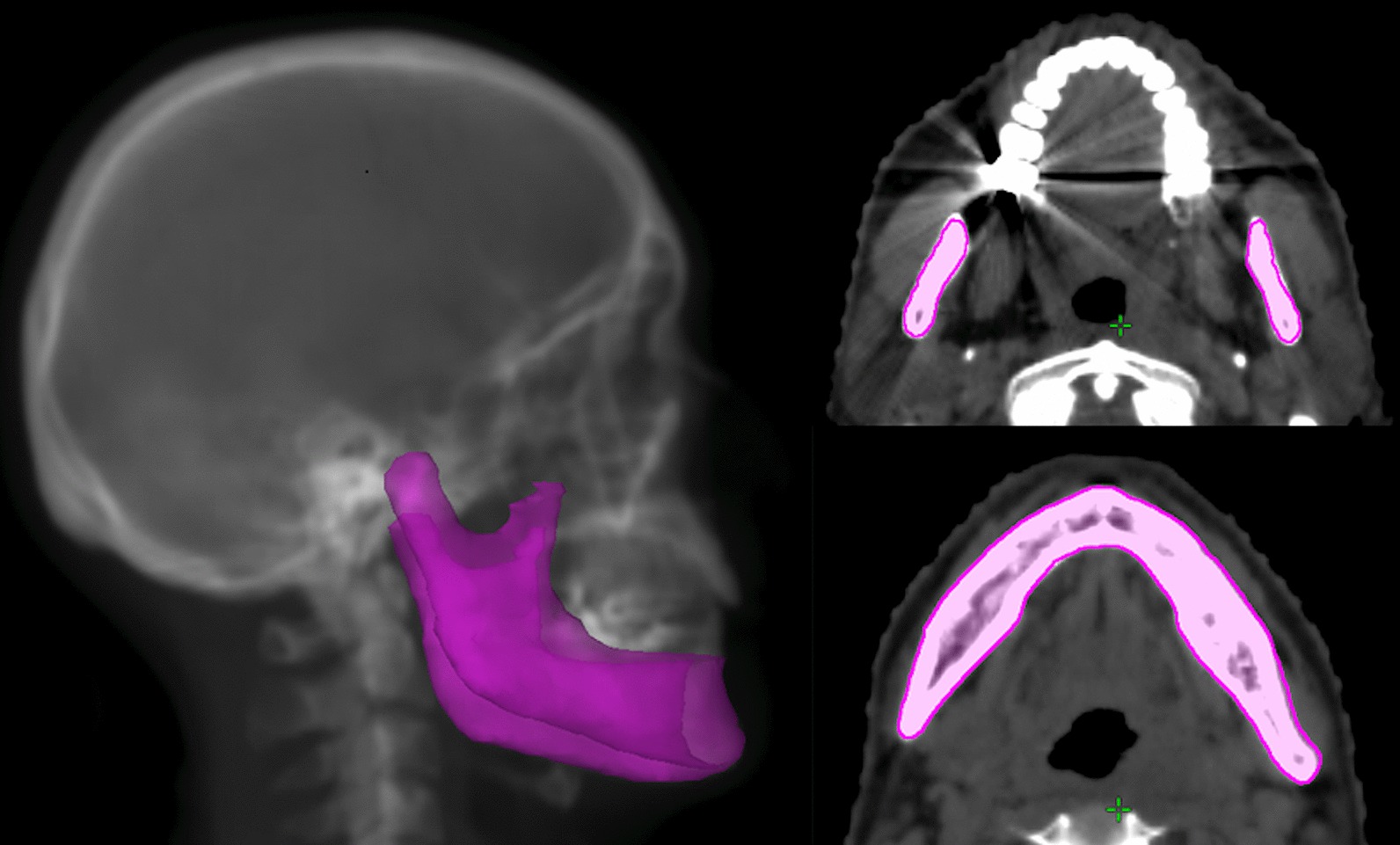
Representative contouring of the jaw. The whole jaw was contoured including the body, angle or ramus, symphyseal or parasymphyseal, condylar process/head, and coronoid

The jaw was contoured by one investigator for each plan (Fig. 1), and DVHs were created. The relative volumes of the jaw exposed to doses ranging from 10 to 70 Gy in 10-Gy increments (V10–70) were reviewed as the RT dosimetric parameters. Individual DVH analysis with respect to the location of the ORNJ was also conducted. The maximum dose to the jaw (Dmax), mean dose to the jaw (Dmean), and dose to the 2 cc of the jaw (D2cc) were reviewed for individual DVH analysis. The ORNJ location was reviewed from patients’ medical records. All DVH analyses were conducted using Velocity (Varian Medical Systems, Palo Alto, CA, USA).

### Statistical analysis

To compare patient- and treatment-related characteristics in the groups with and without ORNJ, Fisher’s test was used to compare the distributions of categorical variables, and t tests were used to compare the distributions of continuous variables. The Mann–Whitney U test was used to compare patients in terms of the presence of ORNJ (present vs. absent), RT technique (3D-CRT vs. IMRT), and primary tumor site (oropharyngeal cancer/oral cancer vs. others) in DVH parameters (V10–70). Cut-off points for DVH parameters were selected based on the Akaike information criterion (AIC) for the development of ORNJ [25]. The AIC is based on in-sample fit for the estimation of the likelihood of a model in predicting future values; the best model is the one with the minimum AIC value among all the other models. Univariate and multivariate Cox proportional hazard models were used to assess patient- and treatment-related factors associated with the development of ORNJ. Factors with statistical significance in the univariate analysis were included in the multivariate analysis. The cumulative incidence was estimated using the Kaplan–Meier method, and the log-rank test was used to compare cumulative incidence curves. The follow-up time was calculated from the last date of RT until the most recent follow-up visit at our institution or the date of death. The time to ORNJ development was calculated from the last date of RT until the date of ORNJ occurrence. All statistical analyses were performed using EZR (Saitama Medical Center, Jichi Medical University, Saitama, Japan), which is a graphical user interface for R (The R Foundation for Statistical Computing, Vienna, Austria) [26]. Statistical significance was defined as a *p* value of 0.05 or less.

## Results

### Patient- and treatment-related characteristics

Among the 616 patients included in the study, the median follow-up time was 40 months (range 3–145 months). ORNJ developed in 46 patients (7.5%) at 47 sites of the jaw, and the median time to ORNJ development was 27 months (range 2–127 months). The patient- and treatment-related characteristics of patients with and without ORNJ are summarized in Table 1. ORNJ developed more frequently among patients with a primary tumor in the oropharynx or oral cavity (*p* < 0.0001), in those who underwent post-RT tooth extraction (*p* < 0.0001), and in those receiving concurrent chemotherapy (*p* = 0.0121) (Table 1).

**Table 1:** Patient, tumor, and treatment characteristics

Characteristic	All patients (n = 616) (%)	Non-ORNJ (n = 570) (%)	ORNJ (n = 46) (%)	*P* value
*Patient-related factors*				
Age, years [median (range)]	65 (23–89)	65.5 (23–89)	64.5 (37–82)	0.2460
Sex				
Male	513 (83)	473 (83)	40 (87)	
Female	103 (17)	97 (17)	6 (13)	0.6810
History of smoking				
No	464 (75)	133 (23)	7 (15)	
Yes	140 (23)	426 (75)	38 (83)	0.2710
Missing	12 (2)	11 (2)	1 (2)	
History of alcohol use				
No	150 (24)	139 (24)	11 (24)	
Yes	449 (73)	416 (73)	33 (72)	1.0000
Missing	17 (3)	15 (3)	2 (4)	
Diabetes mellitus				
No	507 (82)	470 (82)	37 (80)	
Yes	101 (16)	93 (16)	8 (17)	0.8350
Missing	8 (1)	7 (1)	1 (2)	
Performance status				
0	180 (29)	161 (28)	19 (41)	
1	286 (46)	272 (48)	14 (30)	
2	83 (13)	76 (13)	7 (15)	
3	13 (2)	13 (2)	0 (0)	0.0968
Missing	54 (9)	48 (8)	6 (13)	
Pre-RT dental evaluation				
No	128 (21)	118 (21)	10 (22)	
Yes	488 (79)	452 (75)	36 (78)	0.8510
Pre-RT tooth extraction				
No	393 (64)	368 (65)	25 (54)	
Yes	223 (36)	202 (35)	21 (46)	0.2020
Post-RT tooth extraction				
No	567 (92)	533 (94)	34 (74)	
Yes	49 (8)	37 (6)	12 (26)	< 0.0001
*Tumor-related factors*				
Tumor site				
OPC	169 (27)	147 (26)	22 (48)	
OC	161 (26)	143 (25)	18 (39)	
HPC	226 (37)	223 (39)	3 (7)	
NPC	42 (7)	40 (7)	2 (4)	
CUP	18 (3)	17 (3)	1 (2)	0.0001
T-stage				
T0–2	370 (60)	338 (59)	32 (70)	
T3–4	246 (40)	232 (41)	14 (30)	0.2110
Nodal status				
Negative	131 (21)	123 (22)	8 (17)	
Positive	485 (79)	447 (78)	38 (83)	0.5790
*Treatment-related factors*				
Pre-RT surgery				
No	378 (61)	354 (62)	24 (52)	
Yes	238 (39)	216 (38)	22 (48)	0.2090
Pre-RT mandible surgery				
No	565 (92)	526 (92)	39 (85)	
Yes	51 (8)	44 (8)	7 (15)	0.0908
Induction chemotherapy				
No	563 (91)	520 (91)	43 (93)	
Yes	53 (9)	50 (9)	3 (7)	0.7870
Concurrent chemotherapy				
No	172 (28)	167 (29)	5 (11)	
Yes	444 (72)	403 (71)	41 (89)	0.0059
RT technique				
3D-CRT	448 (73)	409 (72)	39 (85)	
IMRT	168 (27)	161 (28)	7 (15)	0.0593
Total radiation dose, Gy [median (range)]	69.96 (50–75)	69.96 (50–75)	69.96 (50–70)	0.5210

Fisher’s exact test *p* values are shown for all covariates except for age and total radiation dose (t-test)

ORNJ, osteoradionecrosis of the jaw; RT, radiation therapy; OPC, oropharyngeal cancer; OC, oral cancer; HPC, hypopharyngeal cancer; NPC, nasopharyngeal cancer; CUP, cervical lymph node metastases from an unknown primary site; 3D-CRT, 3-dimensional conformal radiation therapy; IMRT, intensity-modulated radiation therapy

In total, 83% of 47 ORNJ sites were located in the body of the jaw, 43% were located in the angle or ramus, and 11% were identified in the symphyseal or parasymphyseal area; ORNJ was not observed in the condylar process/head or coronoid. In 16 of the ORNJ sites, overlap was observed in the plural subsite of the jaw.

Of the 47 ORNJ sites, bone exposure was observed in 41 (87%), and cutaneous fistula was seen in 15 (32%). Both bone exposure and cutaneous fistula were noted in 10 sites (21%).

CT scans were evaluable for 46 ORNJ sites. On CT, cortical erosion was observed in 38 (83%) sites, loss of spongiosa trabeculation was noted in 32 (70%) sites, and pathological fracture was observed in 12 (26%) sites.

Of the 46 patients with ORNJ, 22 (47%) had CTCAE grade 2, 23 (49%) had grade 3, and two (4%) had grade 4 ORNJ.

Regarding ORNJ treatment, all patients received conservative management, four (9%) received HBO, and 21 (46%) required surgical management. Of the four patients treated with HBO, two also received additional surgical ORNJ management. At the time of analysis, the disease had been cured or stabilized in 42 (91%) of the 46 patients.

Of the 46 patients with ORNJ, one patient (2%) had a history of bisphosphonate treatment.

### Review of radiation dose distributions in the ORNJ specific site

A review of the radiation dose distributions in the 47 ORNJ sites showed that the median Dmax for the ORNJ regions was 68.5 Gy (range 48.4–77.2 Gy), median D2cc was 62.7 Gy (range 10.8–75.3 Gy), and median Dmean was 62.6 Gy (range 24.4–73.3 Gy).

### Dosimetric comparison of the irradiated jaw

The results of the jaw DVH comparisons are summarized in Table 2. Statistically significant differences were observed between patients with and without ORNJ in the V30 to V70 of the jaw using the nonparametric test. The difference at V60 was the most significant (12.16% vs. 35.31%, *p* < 0.0001). On comparing patients treated with 3D-CRT and IMRT, the V40–70 values were significantly higher with 3D-CRT, whereas the V10–20 values were significantly higher with IMRT. On comparing the primary tumor site, all DVH parameters (V10–70) were significantly higher in oral cancer or oropharyngeal cancer cases than in other primary site cancer cases.

**Table 2:** Comparison of the dose–volume histograms

Characteristic		V10 (%)	V20 (%)	V30 (%)	V40 (%)	V50 (%)	V60 (%)	V70 (%)
*ORNJ*								
No	Median	92.90	86.59	76.61	65.07	41.68	12.16	0.03
Yes	Median	93.99	90.29	84.16	73.48	61.37	35.31	3.22
	* p* value*	0.4050	0.2340	0.0148	0.0016	< 0.0001	< 0.0001	0.0041
*RT technique*								
3D-CRT	Median	87.57	81.37	76.07	70.47	52.84	26.62	0.21
IMRT	Median	99.42	92.92	79.39	53.95	27.30	5.33	0.00
	* p* value*	< 0.0001	< 0.0001	0.6300	< 0.0001	< 0.0001	< 0.0001	0.0001
*Primary tumor site*								
OPC/OC	Median	95.04	90.24	83.21	73.87	56.26	32.03	0.00
Others	Median	88.99	82.00	69.43	57.59	32.86	3.98	1.19
	* p* value*	< 0.0001	< 0.0001	< 0.0001	< 0.0001	< 0.0001	< 0.0001	< 0.0001

^*^Mann–Whitney U test

V10, V20, V30, V40, V50, V60, and V70, represent relative volumes of the jaw exposed to 10, 20, 30, 40, 50, 60, and 70 Gy, respectively

ORNJ, osteoradionecrosis of the jaw; RT, radiation therapy; 3D-CRT, 3-dimensional conformal radiation therapy; IMRT, intensity-modulated radiation therapy; OPC, oropharyngeal cancer; OC, oral cancer

### Cut-off DVH parameter values

**Fig. 2:**
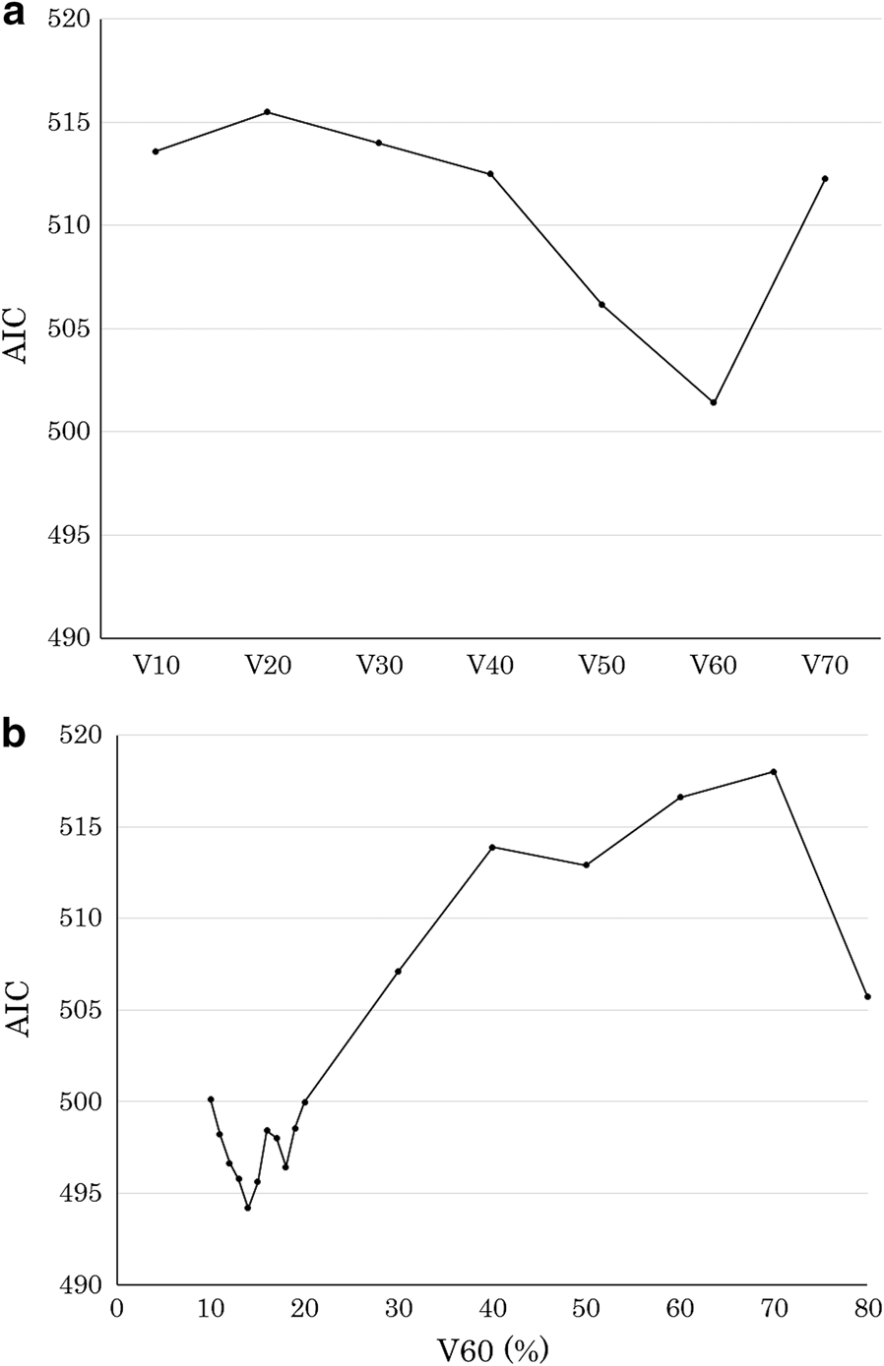
Akaike information criterion (AIC) value of the volume of the jaw receiving between 10 and 70 Gy (**a**), and relationship between AIC and V60 of the jaw (**b**). Vxx, relative volumes of the jaw exposed to xx Gy

To identify optimal cut-off DVH parameter values (V10–70) of the jaw for comparing the ORNJ and non-ORNJ groups, the AIC value was calculated for each of these DVH parameters. The AIC value was the lowest at V60 among V10–70 (Fig. 2a). The AIC value was the lowest when the V60 of the jaw was divided by 14% (Fig. 2b). Therefore, a V60 value of 14% was decided as the cut-off value. The AIC values for V10–70 are shown in Additional file 1.

### Risk factors associated with the development of ORNJ

**Fig. 3:**
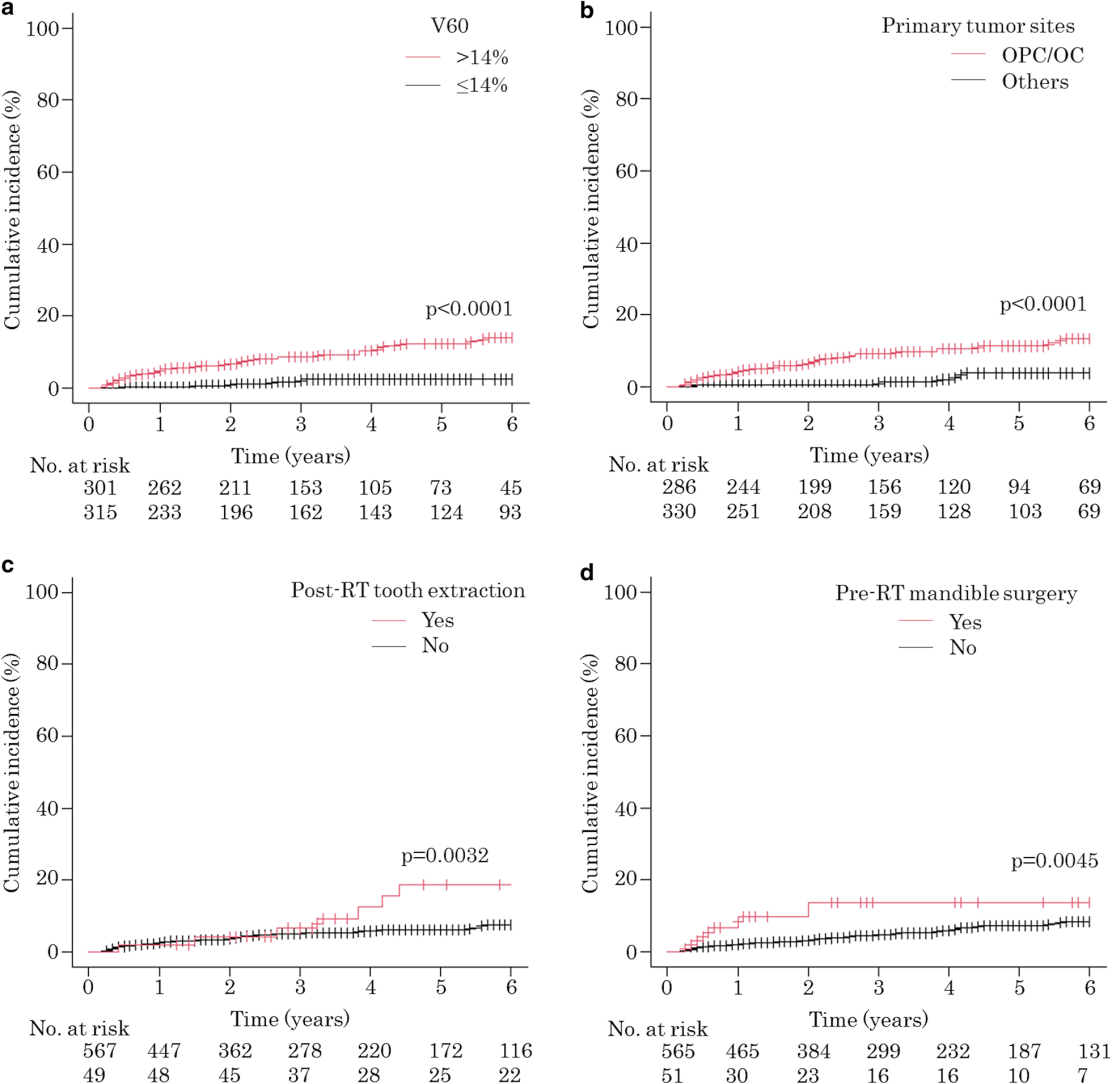
Cumulative incidence curves of ORNJ stratified by V60 (**a**), primary tumor site (**b**), pre-RT mandibular surgery (**c**), and post-RT tooth extraction (**d**). ORNJ, osteoradionecrosis of the jaw; V60, relative volumes of the jaw exposed to 60 Gy; RT, radiation therapy; OPC, oropharyngeal cancer; OC, oral cancer

The results of the univariate and multivariate analyses performed for the clinical factors and DVH parameters for the estimation of their effects on the development of ORNJ are summarized in Tables 3 and 4. The univariate analysis demonstrated a significantly increased risk of ORNJ in association with primary tumor site, pre-RT mandibular surgery, post-RT tooth extraction, and V60 > 14%. Multivariate analyses were performed with primary tumor site (oropharyngeal cancer/oral cancer vs. others), pre-RT mandible surgery, post-RT tooth extraction, and V60 > 14% (Table 4). V60 > 14% was found to be significant (*p* = 0.0065) as a dosimetric parameter in the multivariate models. The primary tumor site was also found to be significant (*p* = 0.0059) as a non-dosimetric factor. The cumulative incidence curves for ORNJ, stratified by V60, primary tumor site, pre-RT mandible surgery, and post-RT tooth extraction are shown in Fig. 3. The 3-year cumulative incidence of ORNJ was 2.5% in patients with V60 ≤ 14% and 8.6% in those with V60 > 14% (*p* < 0.0001). The 3-year cumulative incidence of ORNJ was 9.3% in patients with oropharyngeal cancer or oral cancer and 1.4% in those with other cancers (*p* < 0.0001). The 3-year cumulative incidence of ORNJ was 13.8% in patients with pre-RT mandible surgery and 4.9% in those without (*p* = 0.0045), and 6.6% in patients with post-RT tooth extraction and 5.4% in those without (*p* = 0.032).

**Table 3:** Univariate analysis of the development of osteoradionecrosis of the jaw

Variable	HR	95% CI	*P* value
Age	0.999	0.972–1.026	0.9433
Sex	1.347	0.570–3.180	0.4972
History of smoking	1.489	0.664–3.338	0.3336
History of alcohol use	1.037	0.524–2.052	0.9178
Diabetes mellitus	1.247	0.579–2.682	0.5728
Performance status (0–1 vs. 2–4)	1.608	0.707–3.655	0.2573
Pre-RT dental evaluation	1.280	0.623–2.673	0.5017
Pre-RT tooth extraction	1.469	0.819–2.636	0.1971
Post-RT tooth extraction	2.630	1.350–5.125	0.0045
Tumor site (OPC/OC vs. others)	6.667	2.824–15.74	< 0.0001
T-stage (x, 0–2 vs. 3–4)	0.937	0.497–1.768	0.8410
Lymph node positive	1.408	0.657–3.020	0.3794
Pre-RT surgery	1.713	0.960–3.056	0.0687
Pre-RT mandible surgery	3.053	1.356–6.875	0.0070
Induction chemotherapy	0.748	0.232–2.411	0.6267
Concurrent chemotherapy	2.528	0.997–6.411	0.0507
RT technique (3D-CRT vs. IMRT)	0.886	0.380–2.063	0.7789
Total radiation dose	0.976	0.915–1.041	0.4587
V60 (≤ 14% vs. > 14%)	6.969	2.745–17.700	< 0.0001

HR, hazard ratio; CI, confidence interval; RT, radiation therapy; OPC, oropharyngeal cancer; OC, oral cancer; 3D-CRT, 3-dimensional conformal radiation therapy; IMRT, intensity-modulated radiation therapy; V60, relative volumes of the jaw exposed to 60 Gy

**Table 4:** Multivariate analysis of the development of osteoradionecrosis of the jaw

Variable	HR	95% CI	*P* value
Post-RT tooth extraction	1.769	0.904–3.464	0.0960
Tumor site (OPC/OC vs. others)	3.577	1.443–8.866	0.0059
Pre-RT mandible surgery	1.714	0.753–3.902	0.1988
V60 (≤ 14% vs. > 14%)	3.872	1.460–10.270	0.0065

HR, hazard ratio; CI, confidence interval; RT, radiation therapy; OPC, oropharyngeal cancer; OC, oral cancer; V60, relative volumes of the jaw exposed to 60 Gy

## Discussion

In this study, we evaluated the patient-, tumor-, and treatment-related risk factors associated with ORNJ development and found that V60 > 14% and oropharyngeal or oral cancer were the most significant risk factors.

ORNJ is among the most serious late morbidities observed in patients with HNSCC with curative RT. With the popularization of IMRT planning, dosimetric assessments of the jaw are now performed to reduce the ORNJ risk. In previous studies, a significantly increased risk of ORNJ was observed in patients receiving high doses (> 60–75 Gy) of radiation to the jaw [8, 12–16, 27]. Chen et al. found that a total radiation dose to the primary site of ≥ 75 Gy was an independent factor associated with ORNJ in 1,692 oral cancer patients [8]. Similarly, Chang et al. demonstrated that radiation doses ≥ 70 Gy were predictive of ORNJ [27]. Gomez et al. demonstrated that a maximum dose > 70 Gy and a mean dose > 40 Gy were associated with an increased rate of subsequent dental events and extractions [12], while Aarup-Kristensen et al. reported that the mean dose was significantly associated with ORNJ development [18]. Tsai et al. and Caparrotti et al. suggested that V50 and V60 minimizations were associated with reductions in the rates of ORNJ development in patients with oropharyngeal cancer using a matched case control study [14, 15]. The MD Anderson Head and Neck Cancer Symptom Working Group showed that most patients with ORNJ have a V44 ≥ 42% and V58 ≥ 25% in patients with oropharyngeal cancer using RPA analysis in a matched case control study [16]. In the current study, the values of a wide range of DVH parameters, ranging from V30 to V70, were all significantly higher in the patients with ORNJ than in those without. In our series with a wide spectrum of HNSCC, V60 > 14% was identified as a significant risk factor of ORNJ by both univariate and multivariate analyses. This finding appears to be useful for reducing the risk of ORNJ in daily practice. The cut-off value of V60 appears to be lower than that reported by the MD Anderson Head and Neck Cancer Symptom Working Group (V58 ≥ 25%) due to the differences in the included populations. In the evaluation of the specific ORNJ sites, Owosho et al. demonstrated that 96% of all specific ORNJ sites of the jaw received more than 60 Gy, and the average Dmax was 69.9 Gy (range: 44.3–80.9 Gy) with an average Dmean of 57.4 Gy (range: 28.2–74.6 Gy) [13]. Our results also showed that the median Dmax for the ORNJ regions was 68.5 Gy and the median Dmean was 62.6 Gy, consistent with the findings of Owosho et al.

The primary tumor site is considered a risk factor for the development of ORNJ, as it represents the anatomical localization of the irradiation volume. Our study showed that primary tumor site was among the most important factors related to ORNJ development. Patients with oral cancer or oropharyngeal cancer have a 4.1-fold risk of ORNJ development compared to patients with other cancers. Similar differences in the relative occurrence frequency of the disease, according to the tumor site, have been reported in the literature [19]. In patients with tumors in the oral cavity or oropharynx, the jaw was at least partially included in the high-dose therapeutic area. In fact, we found that the volume of the irradiated jaw was higher in the oral cavity or oropharynx than in the other tumor sites (Table 2). We recommend including the jaw as an organ at risk during IMRT treatment planning, and the use of IMRT optimization objectives to reduce V60 of the jaw below 14% without compromising tumor coverage, especially in the treatment of patients with oropharyngeal and oral cancer.

The incidence of ORNJ has decreased in recent times; this can be attributed to improvements in the rates of identification of relevant risk factors and the RT techniques employed. In the present study, 7.5% of the patients with HNSCC who underwent RT developed ORNJ during the follow-up period, consistent with other recent findings. In general, the incidence of ORNJ varies widely in the literature; this value was as high as 56% in past decades but has dropped to 4–8% in recent times [8, 13, 14, 17–19]. One of the reasons for this consistent decline in the incidence of ORNJ is the use of IMRT, which potentially allows for better normal tissue sparing rates. In a Surveillance, Epidemiology, and End Results-Medicare study, a downward trend in the rate of jaw complications was observed in those receiving IMRT vs. 3D-CRT (14.0% vs. 17.3%, *p* = 0.064) [20]. Similarly, Moon et al. demonstrated significantly lower rates of ORNJ with IMRT use (4.0% vs. 19%, *p* = 0.01) in a study including 252 oropharynx cancer and oral cancer patients [11]. However, Maesschalck et al. found no difference in the ORNJ rates between patients treated with IMRT vs. 3D-CRT (10% vs. 11%); in fact, a higher 3-year cumulative incidence risk was observed in those receiving IMRT (8.9% vs. 4.8%, *p* = 0.03) [21]. Although the present study did not show that IMRT was superior to 3D-CRT for avoiding ORNJ, it is difficult to conclude that there was no difference between the two methods. Several reasons may be considered. One is that follow-up duration and dose per fraction were quite different for both methods. Other reasons are that dose to the jaw was not a dose constraint in IMRT planning and that the reduction of high-dose lesions in the jaw was insufficient in patients treated with IMRT. We believe that use of IMRT contributes to the avoidance of ORNJ; we will adopt the protocol to reduce V60 of the jaw to below 14% in IMRT methods.

Post-RT dental extraction is well-recognized as a patient-related risk factor for ORNJ [28–30]. A systematic review reported that the incidence rate of ORNJ after tooth extraction in irradiated patients was 7% [31]. Wang et al. suggested that post-RT dental extraction was associated with a gradually increasing risk of ORNJ that peaked at 4–5 years [30]. Poor dental health has also been implicated as a risk factor in the development of ORNJ [13]. Whether pretreatment extraction reduces the rate of ORNJ remains unclear. In our study, pretreatment extraction was performed to avoid the risk of ORNJ. In a previous meta-analysis, patients who received pretreatment extraction had a low ORNJ rate of 4.16% [32]. A time period of 10–14 days is recommended between pre-RT extraction and irradiation [32]. Muraki et al. reported that dental interventions, including dental evaluation, prophylactic dental extraction, and good dental hygiene maintenance, both before and after RT have strong effects in the prevention of ORNJ development [33]. Efforts should be driven toward the prevention of post-RT dental extraction both before and after RT.

Pre-RT mandible surgery is another treatment-related risk factor associated with ORNJ [9, 19]. Mandible surgery is often required during the removal of oral cavity tumors adjacent to or infiltrating the mandible. Pre-RT mandible surgery was a risk factor associated with the occurrence of ORNJ in our univariate analysis (*p* = 0.0055); however, its effect was insignificant in the multivariate analysis. Chen et al. reported that patients who underwent segmental mandibulectomy had a higher ORNJ rate compared with those that did not and those who received marginal or hemimandibulectomy [8]. We believe that the extent of surgery and the surgical technique used are also important while considering ORNJ. However, only 51 cases treated with pre-RT mandible surgery were included in this study; further evaluation with a large number of cases is thus necessary.

Along with RT, concurrent chemotherapy offers better local control and overall survival, although it occasionally increases the rates of late toxicity. Whether concurrent chemotherapy increases the risk of ORNJ remains unclear. Reuther et al. observed that ORNJ presented earlier when chemotherapy was used in combination with RT [4]. In contrast, Glanzmann et al. and Wang et al. did not observe increases in the risk of ORNJ with chemotherapy use [30, 34]. In the current study, concurrent chemotherapy had a marginally significant (*p* = 0.051) effect in the univariate analysis. Attention should be paid to the development of ORNJ among patients treated with concurrent CRT.

In our study, the body (83%) and angle or ramus (43%) of the mandible were the most frequent ORNJ sites. Possible reason is that the primary tumor was located close to the body and angle of the mandible in patients with oropharyngeal or oral cancer and close to the high-dose therapeutic area. The blood supply to the mandibular cortex is believed to be an important factor for bone repair. Because the cortex of the body of the mandible is mainly supplied by the periosteal blood flow, insufficient blood supply might be cause of ORNJ occurred at the body of mandible.[35, 36].

This study has several limitations. First, due to its retrospective nature, unmeasured confounding variables and potential selection biases could not be accounted for in our analysis. Second, we did not have data on patient oral hygiene conditions that may contribute to ORNJ development. However, we used data on pre- and post-RT dental extractions as surrogates. Additionally, owing to the study design, each tooth could not be individually assessed by the same clinician in the pre- and post-RT period and standardized for tooth extraction indication. Third, the incidence of post-RT xerostomia was not measured in this study, although it is known that RT exposure to the salivary glands can have an impact on the risk of xerostomia and ORNJ [37]. Fourth, of the patients with ORNJ, only one patient (2%) had a history of bisphosphonate treatment. It may be preferable to exclude the patients with bisphosphonate because there may be an overlap between medication-related osteonecrosis of the jaw (MRONJ) and ORNJ. Because there were only a small number of cases in our cohort (n = 1; 2%) who had treatment with bisphosphonates, we did not exclude the case from our analyses. Finally, the patients examined were all treated at a single institution. Thus, the extent to which our findings can be generalized to other populations may be limited. However, the current study is the first report of the RT dose–volume correlation of the irradiated jaw in a wide spectrum of HNSCC and worthwhile to be considered in daily practice. An additional prospective study with longer follow-up is needed to confirm our suggestion.

## Conclusions

V60 > 14% and oropharyngeal or oral cancer were determined as the most important risk factors for ORNJ development in the univariate and multivariate analyses. These results can prove useful in decision making for the performance of curative RT for head and neck cancers and, subsequently, V60 > 14% appears to be useful in daily IMRT planning for reducing the risk of ORNJ development.

## Supplementary information

**Additional file 1.** Cut-off values according to the Akaike’s information criterion and dose–volume histogram parameters of the jaw.

## Data Availability

The datasets generated and/or analyzed during the current study are not publicly available because they contain personal information but are available from the corresponding author on reasonable request.

## Abbreviations

3D-CRT: 3-Dimensional conformal radiation therapy
ADLs: Activities of daily living
AIC: Akaike’s information criterion
CT: Computed tomography
CTCAE: Common Terminology Criteria for Adverse Events v5.0
D2cc: Dose to the 2 cc of the jaw
Dmax: Maximum dose to the jaw
Dmean: Mean dose to the jaw
DVH: Dose-volume histogram
HBO: Hyperbaric oxygen therapy
IMRT: Intensity-modulated radiation therapy
ORNJ: Osteoradionecrosis of the jaw
RT: Radiation therapy
Vxx: Relative volumes of the jaw exposed to xx Gy
