# Supplementary material for: Risk factors for osteoradionecrosis of the jaw in patients with head and neck squamous cell carcinoma
Source: Radiat Oncol. 2021 Jan 5;16:1. doi: 10.1186/s13014-020-01701-5 (PMC7786900; doi:10.1186/s13014-020-01701-5)
Supplement: Supplementary file 1 — Additional file 1. Cut-off values according to the Akaike’s information criterion and dose–volume histogram parameters of the jaw. [file 13014_2020_1701_MOESM1_ESM.docx]

**Additional file 1** Cut-off values according to the Akaike information criterion and dose-volume histogram parameters of the jaw

|  |  | AIC |
| --- | --- | --- |
| V10 (%) | ≤10, >10 | N/A |
|  | ≤20, >20 | N/A |
|  | ≤30, >30 | N/A |
|  | ≤40, >40 | N/A |
|  | ≤50, >50 | 518.06 |
|  | ≤60, >60 | 514.32 |
|  | ≤70, >70 | 512.59 |
|  | ≤80, >80 | 514.69 |
|  | ≤90, >90 | 516.71 |
| V20 (%) | ≤10, >10 | N/A |
|  | ≤20, >20 | N/A |
|  | ≤30, >30 | N/A |
|  | ≤40, >40 | N/A |
|  | ≤50, >50 | N/A |
|  | ≤60, >60 | N/A |
|  | ≤70, >70 | 518.3 |
|  | ≤80, >80 | N/A |
|  | ≤90, >90 | 513.55 |
| V30 (%) | ≤10, >10 | N/A |
|  | ≤20, >20 | N/A |
|  | ≤30, >30 | N/A |
|  | ≤40, >40 | N/A |
|  | ≤50, >50 | N/A |
|  | ≤60, >60 | N/A |
|  | ≤70, >70 | 511.29 |
|  | ≤80, >80 | 513.57 |
|  | ≤90, >90 | 516.44 |
| V40 (%) | ≤10, >10 | N/A |
|  | ≤20, >20 | 518.18 |
|  | ≤30, >30 | 516.47 |
|  | ≤40, >40 | 514.61 |
|  | ≤50, >50 | N/A |
|  | ≤60, >60 | 515.67 |
|  | ≤70, >70 | 517.8 |
|  | ≤80, >80 | 515.62 |
|  | ≤90, >90 | 515.06 |
| V50 (%) | ≤10, >10 | N/A |
|  | ≤20, >20 | 513.52 |
|  | ≤30, >30 | 508.95 |
|  | ≤40, >40 | 511.01 |
|  | ≤50, >50 | 509.58 |
|  | ≤60, >60 | 510.42 |
|  | ≤70, >70 | 512.21 |
|  | ≤80, >80 | 511.55 |
|  | ≤90, >90 | 517.65 |
| V60 (%) | ≤10, >10 | 500.14 |
|  | ≤ 11, >10 | 498.21 |
|  | ≤ 12, >12 | 496.66 |
|  | ≤ 13, >13 | 495.79 |
|  | ≤ 14, >14 | 494.19 |
|  | ≤ 15, >15 | 495.61 |
|  | ≤ 16, >16 | 498.42 |
|  | ≤ 17, >17 | 498.02 |
|  | ≤ 18, >18 | 496.43 |
|  | ≤ 19, >19 | 498.55 |
|  | ≤20, >20 | 499.98 |
|  | ≤30, >30 | 507.13 |
|  | ≤40, >40 | 513.89 |
|  | ≤50, >50 | 512.91 |
|  | ≤60, >60 | 516.6 |
|  | ≤70, >70 | 518.02 |
|  | ≤80, >80 | 505.75 |
|  | ≤90, >90 | N/A |
| V70 (%) | ≤10, >10 | 517.06 |
|  | ≤20, >20 | 516.00 |
|  | ≤30, >30 | 517.43 |
|  | ≤40, >40 | 514.26 |
|  | ≤50, >50 | 519.41 |
|  | ≤60, >60 | 515.77 |
|  | ≤70, >70 | N/A |
|  | ≤80, >80 | N/A |
|  | ≤90, >90 | N/A |

Abbreviation: AIC, Akaike information criterion.

V10, V20, V30, V40, V50, V60, and V70 represent relative volumes of the jaw exposed to 10, 20, 30, 40, 50, 60, and 70 Gy, respectively.
